# Mutations and altered expression of *SERPINF1* in patients with familial otosclerosis

**DOI:** 10.1093/hmg/ddw106

**Published:** 2016-04-07

**Authors:** Joanna L. Ziff, Michael Crompton, Harry R.F. Powell, Jeremy A. Lavy, Christopher P. Aldren, Karen P. Steel, Shakeel R. Saeed, Sally J. Dawson

**Affiliations:** 1 UCL Ear Institute, University College London, London WC1X 8EE, UK,; 2 Royal National Throat Nose and Ear Hospital, London WC1X 8EE, UK,; 3 Department of ENT Surgery, The Princess Margaret Hospital, Windsor SL4 3SJ, UK; 4 Wellcome Trust Sanger Institute, Hinxton CB10 1SA, UK

## Abstract

Otosclerosis is a relatively common heterogenous condition, characterized by abnormal bone remodelling in the otic capsule leading to fixation of the stapedial footplate and an associated conductive hearing loss. Although familial linkage and candidate gene association studies have been performed in recent years, little progress has been made in identifying disease-causing genes. Here, we used whole-exome sequencing in four families exhibiting dominantly inherited otosclerosis to identify 23 candidate variants (reduced to 9 after segregation analysis) for further investigation in a secondary cohort of 84 familial cases. Multiple mutations were found in the *SERPINF1* (Serpin Peptidase Inhibitor, Clade F) gene which encodes PEDF (pigment epithelium-derived factor), a potent inhibitor of angiogenesis and known regulator of bone density. Six rare heterozygous *SERPINF1* variants were found in seven patients in our familial otosclerosis cohort; three are missense mutations predicted to be deleterious to protein function. The other three variants are all located in the 5′-untranslated region (UTR) of an alternative spliced transcript *SERPINF1-012*. RNA-seq analysis demonstrated that this is the major *SERPINF1* transcript in human stapes bone. Analysis of stapes from two patients with the 5′-UTR mutations showed that they had reduced expression of *SERPINF1-012*. All three 5′-UTR mutations are predicted to occur within transcription factor binding sites and reporter gene assays confirmed that they affect gene expression levels. Furthermore, RT-qPCR analysis of stapes bone cDNA showed that *SERPINF1-012* expression is reduced in otosclerosis patients with and without *SERPINF1* mutations, suggesting that it may be a common pathogenic pathway in the disease.

## Introduction

Otosclerosis is one of the most common causes of hearing impairment among young adults. It is characterized by abnormal bone homeostasis within the otic capsule (1). The remodelling process often involves the stapedio-vestibular joint, and can lead to fixation of the stapedial footplate and a conductive hearing loss that result in clinical otosclerosis. Histological otosclerosis is seen more frequently than clinical otosclerosis; however in these cases, the stapes is not fixed and the diagnosis can only be made post-mortem (2). The age of onset is variable, although hearing loss in clinical otosclerosis typically begins in the third decade and progressively becomes more severe. Hearing loss is bilateral in 70–85% of cases and is usually asymmetrical, developing initially in one ear before progressing to the other (3). Patients choose to undergo surgery to replace all or part of the affected stapes with a prosthetic device which ameliorates the hearing loss to varying degrees. Alternatively, the condition can be managed with hearing-aids (4).

The clinically relevant form of otosclerosis is relatively common in individuals of Indian and European extraction with a reported frequency of 0.3–0.4% in white Europeans; it is much rarer in African, Native American and other Asian populations (4–7). In most individuals, otosclerosis appears to be sporadic and is considered a complex condition with both environmental and genetic factors contributing (7–9). However, otosclerosis also occurs with a strong familial inheritance pattern in up to 50% of cases. The pattern of inheritance in familial otosclerosis is most often consistent with an autosomal dominant mutation that exhibits variable penetrance estimated at 80–90% (7).

**Figure 1.**
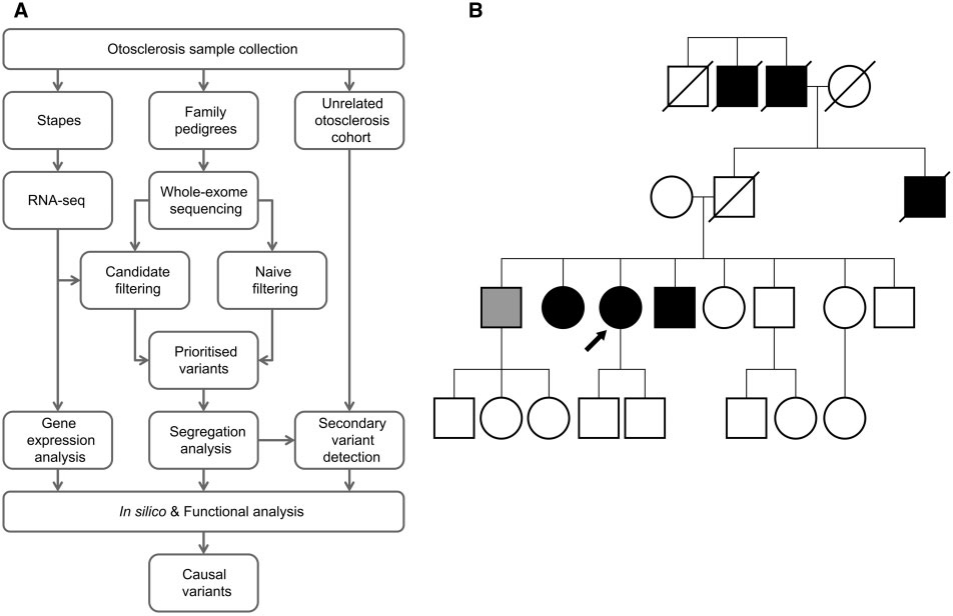
Familial otosclerosis and the next-generation sequencing approach for discovering causal variants. (**A**) Schematic presenting the workflow implementing a combination of whole-exome sequencing, RNA-seq and functional analysis to identify disease-causing variants in familial otosclerosis. (**B**) Pedigree of Family B shows an autosomal dominant inheritance pattern, consistent with familial otosclerosis. A rare heterozygous missense variant, c.601G > A, was identified in *SERPINF1*. Open symbol, clinically unaffected; black symbol, confirmed otosclerosis; light grey symbol, other hearing loss; arrow, proband.

To date, otosclerosis has proved resistant to analysis by conventional genetic techniques. Variable penetrance limits the power of linkage analysis, and so although eight genetic loci have been linked to otosclerosis these chromosomal regions are very large and contain many genes (10–17). As a result, no causal mutations underlying familial otosclerosis have yet been identified limiting progress in therapeutic development (7–9). With the recent advances in sequencing technologies, there have been numerous reports of novel genetic mutations identified using whole-exome sequencing (WES), including the identification of genes involved in rare Mendelian disorders that had previously eluded researchers (18). Furthermore, although more challenging, there is great interest in extending these technologies to common, complex traits (19). Otosclerosis is a heterogeneous disorder in the population but one that often occurs in multiple members of the same family consistent with a monogenic type of inheritance. Hence, a WES approach in these families may provide a powerful discovery tool to reveal the genetic causes and molecular pathways disrupted in otosclerosis. However, because of the frequency of the disease the causal variants are unlikely to be identified by WES alone as frequency filters will have to be set higher than in rare disorders. In this work, we used a combination of WES, RNA-seq and functional analysis to identify disease-causing genes in familial otosclerosis (Fig. 1A). In one family, we detected a rare heterozygous missense variant, c.601G > A, in *SERPINF1* (Serpin Peptidase Inhibitor, Clade F; Fig. 1B). Five additional rare variants were identified in *SERPINF1* from a cohort of 84 familial cases of otosclerosis. We analysed the effect of these mutations and identified *SERPINF1* as the first disease-causing gene in otosclerosis.

## Results

### WES in four families identifies candidate variants for otosclerosis

We sequenced the exomes of 10 individuals from four families with familial otosclerosis, three of European ancestry and one of mixed European and Caribbean ancestry in which the otosclerosis has been inherited via the European ancestral line. Exome capture was performed with an Agilent SureSelect Human All Exon 50 Mb Kit and subject to massively parallel sequencing. An average of 16.5 Gb of sequence was generated per individual as paired-end 100 bp reads. Reads were mapped to the reference sequence (GRCh37_hs37d5) with 96.16% of the bases mapping at >10× coverage. The mean depth of coverage was 178.5-fold with an average of 89 656 variants identified per individual. A filtering strategy was applied to the data in order to prioritize the identified variants (Table 1).

**Table 1.** Variant filtering process applied to whole-exome sequencing data in four families with otosclerosis

Filters	Number of retained variants following application of filter									
	Family A		Family B		Family C		Family D		Average
All variants	88 295		93 972		88 373		87 983		89 656
Quality score > 30	82 689		87 135		82 983		83 058		83 966
Present in all affected individuals in family	58 538		65 275		58 775		55 454		59 511
Exclude variants >0.02 freq 1000G	5924		8339		5844		5865		6493
Exclude variants in 500 Exome Project^a^	4001		—		3910		3925		5044
Exclude synonymous variants	729		1540		695		757		930
Exclude variants in non-coding genes	278		873		279		285		429
Exclude variants in unaffected individuals^b^	—		314		—		—		289


Candidate filters	Cand	Naive	Cand	Naive	Cand	Naive	Cand	Naive	Naive filters
Present in candidate gene list	26	112	26	158	27	94	29	112	Retain deleterious changes
		33		50		31		37	Retain if absent from dbSNP
Exclude homozygous alleles^c^	18	28	—	—	17	29	—	—	Exclude homozygous alleles ^c^
Exclude if on in-house WES database	8	22	12	44	3	21	8	27	Exclude if on in-house WES database
Combined	30		56		23		35	
Prioritized for follow up	4		8		4		7	

Two affected individuals were subject to exome sequencing in families A, B and C and three in Family D. One unaffected individual was also subject to exome sequencing in Family B. After initial filtering (upper panel), variants were filtered in two parallel strategies (lower panel); a candidate gene filter and a robust naive filter based on predicting deleterious variants and rarity. The retained variants were then combined and prioritized for follow up.

^a^Not applied to Family B as the ethnicity of this cohort was not applicable.

^b^Only applied to Family B as only family with unaffected individual.

^c^Not applied to Family B or Family D as both originated from relatively small communities, highlighting a small possibility of the condition exhibiting an autosomal recessive inheritance pattern.

Firstly, variants were filtered within families so that only variants common to all affected individuals were retained. We focused on non-synonymous variants, those affecting splice sites and insertions–deletions (indels) in coding genes. Variants in non-coding genes (with the exception of micro RNAs) and changes within non-coding regions of genes that were not predicted to affect splicing were filtered out. Known variants with a frequency >0.02 were filtered out based on the September 2011 release of 1000 Genomes Project and 500 Exomes Project. This was based on a reported otosclerosis frequency of 0.003–0.004 in Europeans (4,6), of which up to 50% are familial with a dominant inheritance meaning that a familial otosclerosis causal variant would have a frequency of 0.0015–0.002 if homogenous. A filter set 10-fold higher than this is assumed to retain the causal variant especially as there is good evidence that familial otosclerosis is heterogeneous. At this stage, two parallel filtering pipelines were applied (Table 1). These were (i) a robust ‘naive pipeline’ based on retaining variants predicted to be deleterious by either PolyPhen-2 (20) or SIFT (21) and absent from dbSNP134; and (ii) a ‘candidate-gene pipeline’ where only variants in an otosclerosis candidate gene list of 494 genes were retained. The list included genes implicated in otosclerosis from association studies (22–26), gene expression studies (27), genes within linked regions of the genome (10–17,28) and genes known to be involved in other connective tissue disorders that can exhibit an otosclerosis-like hearing loss (29–33). The list also included 176 genes found to be differentially expressed in otosclerotic stapes by an RNA-seq analysis of 12 stapes (J.L. Ziff, J.A. Lavy, S.R. Saeed and S.J. Dawson, manuscript in preparation). Finally, for both pipelines variants were removed if they were also present in a previous in-house exome sequencing project of 20 individuals without otosclerosis.

The remaining variants from both pipelines were annotated and 23 prioritized for follow-up firstly in segregation analysis (Supplementary Material, Table S3). Prioritization was based on a number of factors including GERP score, predicted effect of the variant, expression in otosclerotic and control stapes and known biological role. Fourteen of 23 variants were ruled out due to lack of familial segregation leaving nine candidate variants in the following genes: *ANKS1A* (c.1291C > A), *SERPINF1* (c.601G > A), *VPS53* (c.107C > G), *TRIM17* (c.915C > G), *COL1A2* (c.808G > A), *FZD2* (c.655C > T), *GNGT1* (c.82C > A), *mir183* (n.81G > T) and *ZNF225* (c.1698A > T).

### Additional *SERPINF1* variants identified in a familial otosclerosis cohort

The remaining nine segregated variants were genotyped in 53 further unrelated individuals with a family history of otosclerosis by Sanger sequencing of the exon and exon/intron boundaries. None of the variants listed above was detected in the cohort of 53 unrelated cases of familial otosclerosis. However, a second rare non-synonymous variant (c.441G > C) was found in the *SERPINF1* gene in two unrelated individuals. Exon 5 of *SERPINF1* was subsequently sequenced in a further 31 unrelated individuals with a family history of otosclerosis identifying a novel 3 bp deletion (c.440-40_440-38delTCG) in one individual. None of these variants was found in 175 control samples sequenced. Further sequencing of all coding exons and intron–exon junctions in *SERPINF1* in the otosclerosis cohort revealed three additional rare non-synonymous mutations in exon 3 and exon 4 of *SERPINF1* (c.167C > G, c.331G > A, c.392C > A). In summary, six rare heterozygous variants were identified in seven unrelated patients in the *SERPINF1* gene (see Table 2). Allele frequencies of the *SERPINF1* variants identified were much greater in the unrelated otosclerosis cohort compared with their reported frequencies in the variant databases, 1000 Genomes and NHLBI Exome Sequencing Project.

**Table 2.** Rare variants in *SERPINF1* identified in a cohort of unrelated individuals with familial otosclerosis

						Transcript *SERPINF1-001*			Transcript *SERPINF1-012*		
Sample ID	Identifier	Otosc (*n*)	Controls	1000G	NHLBI	Nucleotide exchange^a^	Exon	Protein effect	Nucleotide exchange^b^	Exon	Protein effect
OTSC147	rs76119062	0.9% (1)^c^	–	n.d.	0.01%	c.167C>G	3	p.Ala56Gly	–	–	–
OTSC140	None	0.9% (1)^c^	–	n.d.	n.d.	c.331G>A	4	p.Asp111Asn	–	–	–
OTSC383	rs148005190	0.9% (1)^c^	–	n.d.	0.08%	c.392C>A	4	p.Ala131Asp	–	–	–
OTSC319	None	0.6% (1)^d^	n.d.	n.d.	n.d.	c.440-40_440- 38delTCG	–	–	c.-202_-200 delTCG	1	5′-UTR
OTSC354; OTSC161	rs138341386	1.1% (2)^d^	n.d.	n.d.	0.03%	c.441G>C	5	p.Lys147Asn	c.-161G>C	1	5′-UTR
OTSC134	rs137997656	0.6% (1)^d^	n.d.	n.d.	n.d.^e^	c.601G>A	5	p.Asp201Asn	c.-1G>A	1	5′-UTR

Allele frequencies of the variants are shown in the unrelated otosclerosis cohort, Otosc; a control cohort (*n* = 175), Controls; the 1000 Genomes Project (Phase 3 European, *n* = 503), 1000G; and the NHLBI Exome Sequencing Project (ESP6500 European-Americans, *n* = 4300), NHLBI. Effects on the amino acid structure of *SERPINF1-001* and *SERPINF1-012* are shown. n.d., not detected.

^a^GenBank: NG_028180.1; Ensembl Transcript: ENST00000254722.

^b^Ensembl Transcript: ENST00000573763.

^c^*n* = 57.

^d^*n* = 88.

^e^The individual with c.601G > A is of mixed European and Caribbean origin; the frequency of c.601G > A in NHLBI ESP6500 cohort Americans of African extraction is 0.3% (*n* = 2203).

**Figure 2.**
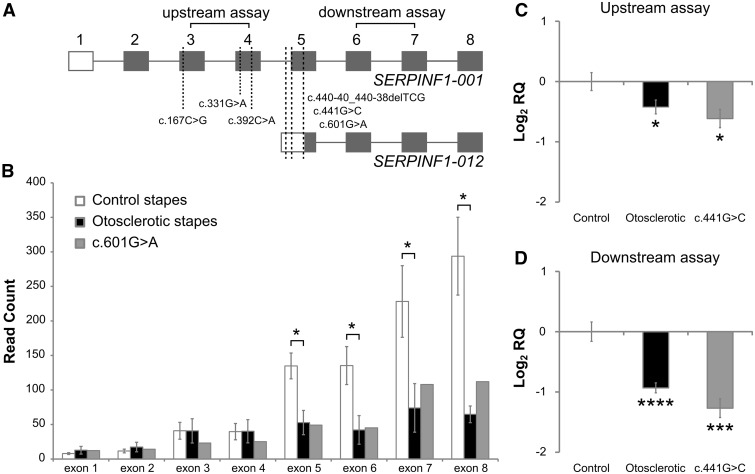
*SERPINF1* transcript expression in control and otosclerotic stapes. (**A**) *SERPINF1-001* and *SERPINF1-012* transcripts (not to scale) with the identified variants indicated by dashed lines. Open box, 5′-UTR; grey box, coding sequence. The positions of the Taqman® gene expression assays used in RT-qPCR are also indicated. (**B**) The average read count of *SERPINF1* exons is shown in eight otosclerotic and four control stapes subjected to RNA-seq analysis, the read count of the stapes from the proband of family B with a c.601G > A mutation is also shown separately. Data indicate a significant difference in expression between otosclerotic and control stapes of exons 5–8 but not exons 1–4. (*P < 0.05. Error bars indicate standard error of mean. A student’s two-tailed *t*-test was performed.) (**C, D**) RT-qPCR data show the average relative expression levels of (C) upstream (exons 3–4) and (D) downstream (exons 6–7) *SERPINF1* exons in stapes from: control individuals (*n* = 5), otosclerosis patients (*n* = 75) and stapes from one individual with a *SERPINF1* mutation (c.441G > C). Expression levels were calculated relative to 18s RNA endogenous control and to levels in controls (calibrator sample). Levels of *SERPINF1* are significantly reduced in the otosclerotic stapes and in c.441G > C for both assays, a greater reduction being detected in downstream exons. Error bars indicate 95% confidence intervals (*P < 0.05; ***P < 0.0005; ****P < 0.00005 in a Student’s two-tailed *t*-test performed on ΔΔCt values compared with control sample).

The c.167C > G, c.331G > A and c.392C > A missense variants cause p.Ala56Gly, p.Asp111Asn and p.Ala131Asp changes, respectively, and are all predicted to be damaging by both PolyPhen-2 (20) and MutationTaster (34) (Table 3). The remaining variants c.441G > C, c.601G > A and c.440-40_440-38delTCG are predicted to have benign effects on the *SERPINF1-001* transcript (ENST00000254722). However, all three variants are located within the 5′-untranslated region (UTR) of the shorter alternatively spliced *SERPINF1-012* transcript (ENST00000573763) which contains exons 5–8 only (see Fig. 2A).

**Table 3.** Summary of *in silico* and functional analysis of identified *SEPRINF1* variants

Transcript *SERPINF1-001*					Transcript *SERPINF1-012*					
Nucleotide exchange^a^	Exon	Protein effect	PolyPhen-2 (HumDiv Score)	MutationTaster (probability)	Nucleotide exchange^b^	Exon	Protein effect	Mfold	TF binding	Luciferase activity
c.167C>G	3	p.Ala56Gly	Probably damaging (0.988)	Disease causing (0.999)	—	—	—	—	—	—
c.331G>A	4	p.Asp111Asn	Possibly damaging (0.879)	Disease causing (0.999)	—	—	—	—	—	—
c.392C>A	4	p.Ala131Asp	Probably damaging (0.969)	Disease causing (0.707)	—	—	—	—	—	—
c.440-40_440- 38delTCG	—	—	—	Polymorphism (0.999)	c.-202_-200 delTCG	1	5′-UTR	Affected	Affected	Affected
c.441G>C	5	p.Lys147Asn	Benign (0.135)	Disease causing (0.995)	c.-161G>C	1	5′-UTR	No change	No change	Affected
c.601G>A	5	p.Asp201Asn	Benign (0.000)	Polymorphism (0.999)	c.-1G>A	1	5′-UTR	No change	Affected	Affected

^a^GenBank: NG_028180.1; Ensembl Transcript: ENST00000254722.

^b^Ensembl Transcript: ENST00000573763.

### *SERPINF1* transcript expression in control and otosclerotic stapes

To determine whether the mutations in *SERPINF1-012* 5′-UTR may play a role in otosclerosis, we examined the read counts for each *SERPINF1* exon in the RNA-seq data from stapes bone to assess alternatively spliced isoform expression (Fig. 2B). In control stapes (*n* = 4), it was found that the average read counts in *SERPINF1* exons were greater for exons 5–8 than exons 1–4. This suggests that the *SERPINF1-012* transcript, which contains exons 5–8 only, is the major *SERPINF1* isoform in the stapes. Additionally, analysis of stapes tissue from the proband in Family B with the c.601G > A variant in the 5′-UTR of *SERPINF1-012* indicated that levels of this transcript are reduced in this individual suggesting that they do affect expression. Moreover, the average read counts for *SERPINF1* exons 5–8 were significantly reduced in otosclerotic stapes (*n* = 8) compared with control stapes (*P* < 0.05; Fig. 2B), whereas no significant differences were observed for the average read counts in exons 1–4 between control and otosclerotic stapes.

Expression of *SERPINF1* transcripts was further investigated in a second, larger sample of stapes using RT-qPCR. This constituted stapes suprastructure from 75 unrelated affected individuals, 5 control individuals and 1 affected individual with the c.441G > C mutation in the 5′-UTR of *SERPINF1-012*. RT-qPCR was used to determine the relative expression of upstream (assay spanning exons 3 and 4) and downstream exons (assay spanning exons 6 and 7), which relate to *SERPINF1-001* and *SERPINF1-012* transcripts, respectively (see schematic in Fig. 2A). A large 2.4-fold reduction was found for the downstream assay in the stapes from the individual with the c.441G > C mutation compared with controls suggesting that this *SERPINF1-012* transcript is affected by the variant in the 5′-UTR of this transcript (*P* < 0.0005; Fig. 2D). In addition, we found that the level of *SERPINF1* mRNA is significantly reduced in the general otosclerosis sample for both assays compared with controls (Fig. 2C and D). This reduction is greater for the downstream exons (1.9-fold, *P* < 0.00005) contained within *SERPINF1-012* than for upstream exons (1.3-fold, *P* < 0.05), consistent with RNA-seq data.

### Investigating the effect of *SERPINF1-012* 5′-UTR variants on expression

Annotation of non-coding variants and prediction of their effects are still limited, meaning that the evaluation of effect of the 5′-UTR variants on *SERPINF1* requires further investigation. Data from RT-qPCR and RNA-seq indicated that two of the mutations in the 5′-UTR reduce *SERPINF1-012* expression in stapes removed from these individuals. 5′-UTR variants can affect regulation of both transcription and translation, either by affecting the binding of transcription factors or by altering the stability of the mRNA through changes in secondary structure. *In silico* analysis using a transcription factor prediction tool, MatInspector (35), predicted that the three *SERPINF1-012* 5′-UTR variants are within various transcription factor binding sites (see Supplementary Material, Table S4). Most notably, both the c.440-40_440-38delTCG and c.601G > A variants were predicted to create binding sites for transcription factors involved in bone regulation; PAX3 (c.440-40_440-38delTCG), RBPJ and NFYA (c.601G > A) (36–38). We also investigated the effects of the 5′-UTR variants on RNA folding using Mfold (39), which predicts RNA structure based on free energy minimization. RNA structures of the 5′-UTR alone and the full length *SERPINF1-012* mRNA were predicted (Supplementary Material, Fig. S1). The c.440-40_440-38delTCG variant showed a marked difference in both the 5′-UTR and full mRNA folding predictions.

**Figure 3.**
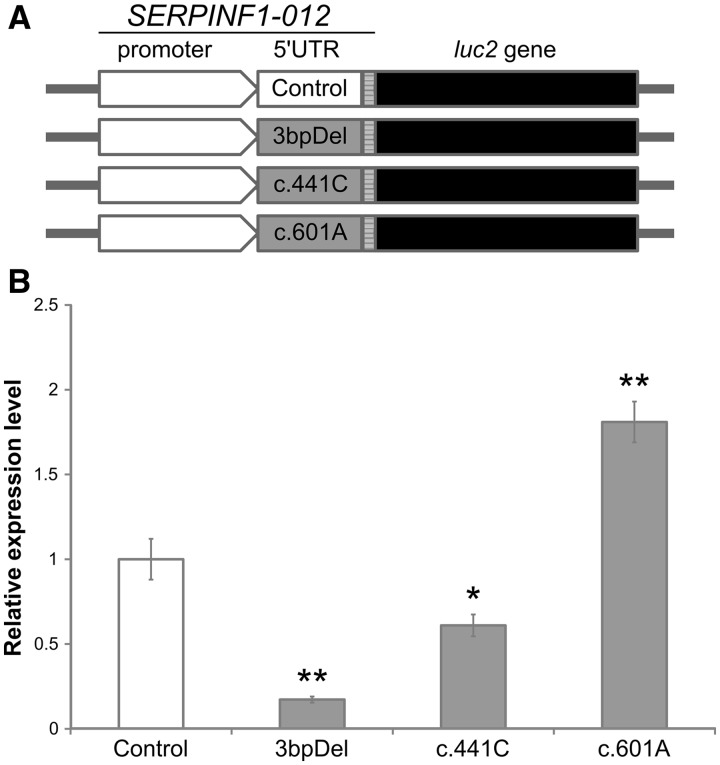
Mutations in the 5′-UTR influence translational efficiency of the *SERPINF1-012* transcript. (**A**) Fragments containing the wild-type or mutant 5′-UTRs of the *SERPINF1-012* transcript were cloned into the pGL4.10 expression vector and transfected into MG-63 cells. (**B**) For the luciferase assay wild-type values were set to 1, to which mutant values were normalized. These results are the average of 12 repeats in four independent experiments. The c.440-40_440-38delTCG (3bpDel) and c.441G > C mutations show a significant reduction in translational activity, whereas the c.601G > A mutation displays a significant increase. Error bars indicate standard error of mean (*P < 0.05; **P < 0.01 in a Student’s two-tailed *t*-test versus control data).

To confirm whether the 5′-UTR mutations have effects on protein production, luciferase constructs containing wild-type and mutant 5′-UTR sequences were tested in MG-63 cells (Fig. 3A). Compared with the wild-type construct, the c.440-40_440-38delTCG and c.441G > C alleles showed a significantly reduced level of luciferase activity (Fig. 3B), indicating that these variants will likely reduce the translational efficiency of the *SERPINF1-012* transcript. The c.601G > A allele also had a significant influence on luciferase activity; however, a significant increase in translational activity was observed.

## Discussion

Despite its high prevalence and disease burden, the impact of hearing impairment on quality of life is often underestimated. Hearing loss can lead to social isolation and depression, and is associated with cognitive decline (40–44). Otosclerosis is one of the most common causes of hearing impairment in young adults and has a significant detrimental effect upon patients (45). Despite the importance of the disease, the genetic pathways and aetiology of otosclerosis remain poorly understood. Using a WES approach we identified a rare *SERPINF1* mutation in a family affected by an autosomal dominant otosclerosis. Five additional mutations were identified in six individuals across a cohort containing 84 unrelated individuals with a family history of otosclerosis meaning that 8.0% of familial otosclerosis patients had a rare *SERPINF1* mutation. Three of these variants have not been reported in the relevant ethnic groups of the 1000 Genomes and NHLBI exome sequencing project and the remaining variants are extremely rare in the same studies (see Table 2). Of the six mutations identified, three are predicted to be deleterious to the protein function of the major *SERPINF1* transcript (*SERPINF1-001*; Table 3). The remaining three mutations are predicted to have benign effects on *SERPINF1-001*; however, they lie within the 5′-UTR of an alternative transcript, *SERPINF1-012*. Assessing the contribution of non-coding variants to disease is limited by lack of knowledge of the effect of such variants. Here, we utilized transcriptomic data in the form of RNA-seq from the relevant tissue to reveal that the alternatively spliced *SERPINF1-012* transcript is the major transcript found in stapes bone, indicating that it plays a role in this tissue. Furthermore, both c.441G > C and c.601G > A 5′-UTR variants result in reduced levels of the exons present in this transcript in stapes bone of the affected patient, suggesting that they alter expression of this transcript. Stapes was unavailable from the patient with the c.440-40_440-38delTCG mutation. The impact of these three 5′-UTR variants was further assessed using *in vitro* luciferase assays, showing that all three *SERPINF1-012* 5′-UTR mutations had a significant impact on the translation of the reporter gene (see Fig. 3), highlighting the functional importance of the identified mutations. Whether these effects are mediated by altering transcription or translation remains to be demonstrated but it is intriguing that two of the variants are predicted to create binding sites for transcription factors known to be involved in regulating bone turnover; PAX3, RBPJ and NFYA (36–38). PAX3 and RBPJ have both been shown to play inhibitory roles in bone formation, whereby persistent PAX3 expression inhibits BMP-induced osteogenesis (36) and activation of RBPJ inhibits TNF-induced osteoclastogenesis (37). In osteosarcoma cells, NFYA has been shown to stimulate transcription of bone sialoprotein, a major protein in the extracellular matrix of bone (38). This raises the possibility that the pathological mechanism is aberrant transcriptional regulation of *SERPINF1-012* leading to dysregulated bone regulation.

The most surprising observation in our study is that *SERPINF1-012* expression is also reduced in stapes from a heterogeneous cohort of 75 unrelated otosclerosis patients (Fig. 2D). This heterogeneous set of patients included both sporadic and familial cases, which suggests that reduction of this transcript may be a common pathway in the pathology underlying otosclerosis. Therefore, other genes in this pathway may also represent good candidates for mutation in other familial cases.

*SERPINF1* encodes PEDF (pigment epithelium-derived growth factor), a collagen-binding protein that is highly expressed in collagen-rich tissues including bone, cornea and cartilage. PEDF is a potent inhibitor of angiogenesis (46), and the collagen-binding property of PEDF has been elucidated to be important for its anti-angiogenic activity (47). Interactions between angiogenic and osteogenic pathways are known to be essential in bone formation, repair and remodelling (48), highlighting PEDF as a good biological candidate in otosclerosis. Additionally, PEDF is known to bind to heparan sulphate, a glycosaminoglycan which regulates transforming growth factor beta (TGF-β) signalling by modulating the assembly of latent TGF-β1 (49). TGF-β1 is a polypeptide abundant in the bone matrix that is known to be a potent stimulator of osteoblastogenesis and inhibitor of osteoclastogenesis, for which reason it has been repeatedly proposed as an otosclerosis candidate gene (50–52). Various members of this family including TGF-β1 and bone morphogenetic proteins BMP2 and BMP4 have been associated with otosclerosis in some candidate gene association studies but not all (22,23,52–54); however, a precise role for this gene family in development of otosclerosis has not been defined. The identification of *SERPINF1* mutations in patients with familial otosclerosis in this study and the altered stapes expression observed in the gene that encodes PEDF provide a new link between heparan sulphate and modulation of TGFβ signalling in otosclerosis. Furthermore, heparan sulphate has been shown to compete with PEDF in interactions with collagen, due to overlapping binding sites (55), highlighting a possible functional relationship between collagen, PEDF and heparan sulphate during angiogenesis. Some of the residues involved in heparan binding are absent from the shorter alternatively spliced *SERPINF1-012* transcript, which could have a significant impact on its role in angiogenesis and bone regulation in the stapes compared with *SERPINF1-001* in other bone.

Mutations in *SERPINF1* are already known to underlie one recessive form of the connective tissue disorder osteogenesis imperfecta (OI) type VI (MIM: 613982). OI is a brittle bone disorder caused by mutations in *COL1A1* or *COL1A2* in over 90% of cases; with mutations in many other genes, including *SERPINF1*, responsible for the other 10% of cases. To date 20 unique sequence variants in *SERPINF1* have been identified in OI type VI patients (56–65). Most of the reported variants are nonsense or frameshift mutations that are expected to cause mRNA instability due to nonsense-mediated decay, leading to complete loss of PEDF expression (60). The three *SERPINF1* in-frame deletions or insertions identified in OI lead to retention or degradation within cellular compartments and thereby interfere with PEDF secretion (66). Although it is expected that a loss of PEDF results in production of under-mineralized bone, the mechanism by which this occurs is unknown. In bone, as PEDF binds to collagen with high affinity and is actively expressed in osteoblastic regions of active bone formation, it is possible that PEDF plays an important functional role in bone matrix remodelling (67). Some patients with OI experience conductive hearing loss similar to that of otosclerosis. These similarities have previously led researchers to postulate that otosclerosis and OI may have a common aetiology (68). Therefore, given *SERPINF1*’s known role in bone regulation these data suggest *SERPINF1* is an excellent candidate for an otosclerosis gene. Importantly, all of the otosclerosis patients with *SERPINF1* mutations display no OI and have not reported a disproportionate amount of broken bones. One patient reported having osteoporosis and rheumatoid arthritis.

**Figure 4:**
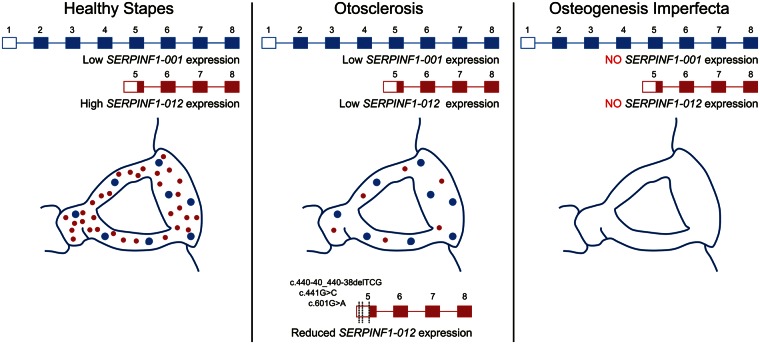
Proposed role of *SERPINF1-012* expression in otosclerosis pathophysiology. The alternatively spliced *SERPINF1-012* transcript (shown in red) is the major transcript found in healthy stapes bone, whereas *SERPINF1-001* (shown in blue) has a lower level of expression. Reduced expression of *SERPINF1-012* expression by mutation in familial cases or by other means in sporadic otosclerosis leads to localized stapes bone dysregulation and otosclerosis. Loss of *SERPINF1-001* expression due to frameshift and nonsense mutations in osteogenesis imperfecta causes systemic bone dysregulation.

In contrast to OI, none of the mutations described here in the otosclerosis cohort are nonsense or frameshift mutations, so it is unlikely that they would lead to nonsense mediated decay. The variants identified were found to have a more subtle effect on SERPINF1 function. The three missense variants predicted to be deleterious to the protein function of SERPINF1 (c.167C > G, c.331G > A and c.392C > A) would be expected to only cause a partial loss of PEDF function rather than the complete loss of PEDF expression seen in OI patients. The c.440-40_440-38delTCG, c.441G > C and c.601G > A variants identified in the 5′-UTR of *SERPINF1-012* were shown to have a significant impact on the translational efficiency of this transcript; therefore, it is possible that these variants primarily affect an alternatively spliced transcript which is critical for stapes maintenance but redundant in other bone (Fig. 4). This could explain the phenotypic differences seen between OI and otosclerosis and would support the suggestion that they have a common aetiology.

The results from this study indicate that WES is a useful tool for investigating disease-causing variants in families exhibiting autosomal dominant inheritance of otosclerosis. The step-wise variant prioritization process has been effective at narrowing down the large number of potential disease-causing variants from an average of 89 656 variants per family to a small pool of those that are most likely to be involved in the disease process. Ultimately, our results demonstrate the power of an exome sequencing approach over linkage analysis in familial cases of a relatively common disorder, even in the presence of variable penetrance and heterogeneity. It also shows the value of transcriptome data in the filtering and prioritization of variants, highlighting *SERPINF1* as the first disease-associated gene to be identified in otosclerosis.

## Materials and methods

### Patient recruitment

Individuals with a confirmed diagnosis of otosclerosis were recruited from the Royal National Throat Nose and Ear Hospital, London, UK and The Princess Margaret Hospital in Windsor. The study was approved by the London Bloomsbury NRES Ethics committee (11/LO/0489) and patients were recruited by informed consent. From this cohort, a sub-cohort of individuals with evidence of familial otosclerosis was identified based on patient questionnaire data of family history (defined as two or more relatives with otosclerosis). Blood or saliva samples (Oragene® Saliva Kit, DNA Genotek) were obtained for genomic DNA isolation by standard methods. For those patients undergoing stapedotomy surgery to remove the stapes superstructure, the tissue was retained for gene expression studies. Control stapes were obtained from individuals where the stapes was removed as part of surgery for a number of other indicators including head trauma, glomus tumour and during total petrosectomy.

### WES and filtering

WES was performed at the Wellcome Trust Sanger Institute on genomic DNA isolated from each of the 10 individuals selected from four families with familial otosclerosis. The sequencing data have been deposited at the European Genome-phenome Archive (EGA; EGAS00001000156). After target enrichment using Agilent SureSelect Human All Exon V3 kit the whole-exome DNA library from each participant was sequenced using Illumina HiSeq 2000 with 100 bp paired-end reads and mapped to the reference human genome (GRCh37_hs37d5) based on the sequence alignment. Both single-nucleotide variants and indels were identified using SAMtools (69), GATK (70) and Dindel (71). A mean of 16.5 Gb of sequence was generated per sample with an average of 96.16% of the bases mapping to the reference genome at a coverage of at least 10×. The mean depth of coverage was 178.5×. Of all the variants mapped, 93.7% met a quality score threshold of 30. The filtering strategy applied to the data is described in Table 1 and in the main text.

### Segregation analysis and mutation detection

All variants prioritized for follow-up were confirmed by Sanger sequencing in the relevant family. Genotyping for segregation analysis was performed by Sanger sequencing. In segregation analysis, variants were excluded if they were present in more than one unaffected individual above the age of onset of otosclerosis in that family. Variants were also excluded if they were not present in all individuals with otosclerosis. Sanger sequencing was used for genotyping in the unrelated familial otosclerosis cohort. PCR primers used to amplify the *SERPINF1* exons are shown in Supplementary Material, Table S1.

### RNA-seq and RT-qPCR analysis

Human stapes were preserved in AllProtect™ Solution (Qiagen) and stored at –80 °C. Stapes were homogenized in QIAzol Lysis reagent and RNA purified using the RNeasy lipid tissue mini kit (Qiagen) according to the manufacturer’s protocol, including an on column DNase digest. RNA was reverse transcribed into cDNA by Clontech SMARTer™ kit for RNA-seq and by Omniscript Reverse Transcriptase Kit for RT-qPCR. The integrity of the cDNA for RNA-seq was confirmed on an Agilent Bioanalyzer 2100 before sending to Otogenetics Corp (Norcross, GA, USA) where cDNA fragmentation was performed on the samples and cDNA libraries constructed before 100 bp paired-end RNA sequencing using Illumina HiSeq 2000 was carried out. Paired-end 100 nt reads were aligned to human genomic assembly hg19 and visualized on the DNAnexus platform (Mountain View, CA, USA). Taqman® gene expression assays were performed on human otosclerotic and control cDNA samples. RT-qPCR was conducted using an SDS7500 real time PCR machine (Applied Biosystems) with primer/probe pairs obtained from Applied Biosystems. *SERPINF1* assays spanning exons 3–4 (Assay ID: Hs011006934_m1) and exons 6–7 (Assay ID: Hs011006937_m1) were performed with eukaryotic 18s RNA as an endogenous control. Relative quantification of *SERPINF1* was calculated relative to a calibrator sample (control stapes) using the ΔΔCt method.

### *In silico* analysis of 5′-UTR variants

MatInspector (35), a transcription factor prediction tool, was used to identify potential transcription factor binding sites in the region where the variants in the 5′-UTR of the *SERPINF1-012* transcript were found. The matrix score calculates a match between the sequence and the matrix, ranging from 0 to 1, with 1 indicating an exact match. Putative RNA folding structures were predicted using Mfold (39) with standard settings.

### Cloning of *SERPINF1-012* 5′-UTR variants

The Gibson assembly cloning method (72) was used to insert the DNA fragments of three *SERPINF1-012* 5′-UTR variants into pGL4.10 vector separately, as well as a wild-type control. Gibson assembly primers for *SERPINF1-012* fragments were synthesized (Supplementary Material, Table S2), and inserts were prepared by PCR amplification using the Q5 High-Fidelity PCR kit (New England Biolabs) and gDNA as a template. PCR products were used in DNA assembly reaction with PCR-linearized pGL4.10 vector using Gibson Assembly Cloning kit (New England Biolabs). Each assembly reaction contained approximately 150 ng of insert and 50 ng of the expression vector and incubated at 50 °C for 1 h following the manufacturer’s protocol. After the assembly reaction, the reaction mix was transformed into NEB 5-alpha competent *E. coli* strain (New England Biolabs). After an overnight growth at 37 °C, the pGL4.10 plasmids containing respective inserts were extracted using a QIAprep Spin Miniprep Kit (Qiagen) and constructs were verified by Sanger sequencing.

### Transfection and luciferase activity assay

To measure the translational activity of the 5′-UTR variants of *SERPINF1-012*, dual-luciferase reporter assays were conducted in MG-63 cells. The human osteosarcoma MG-63 cell line was obtained from Dr Vehid Salih at the Eastman Dental Hospital, University College London. MG-63 cells were cultured in Dulbecco’s modified Eagle medium (DMEM; Invitrogen) and were maintained at 37 °C in a 5% CO_2_ incubator. Medium was supplemented with glutamax, 10% foetal bovine serum and 1% penicillin/streptomycin. MG-63 cells (1 × 10^5^) were seeded on 6-well plates and transfected 24 h later using the calcium phosphate method (73). A total of 270 ng plasmid DNA (250 ng of pGL4.10 constructs and 20 ng phRL-null) was used for each transfection. After incubation for 16 h, cells were exposed to glycerol shock condition with 2 ml of 15% glycerol containing DMEM for 2 min 30 s. Subsequently, the mixture was aspirated and replaced with fresh DMEM supplemented with glutamax, 10% foetal bovine serum and 1% penicillin/streptomycin. Cells were harvested 48 h after transfection, and luciferase activities were measured with the Dual-Luciferase® Reporter Assay System (Promega) and a single-tube luminometer (Turner BioSystems). Luciferase activities shown in figures are the mean of 12 transfections in four independent experiments.

## Web resources

The URLs for data presented herein were last accessed on February 23, 2016 and are as follows:

1000 Genomes, http://www.1000genomes.org/

Ensembl, http://www.ensembl.org/

European Genome-phenome Archive (EGA), https://www.ebi.ac.uk/ega/

MatInspector, https://www.genomatix.de/matinspector

Mfold, http://unafold.rna.albany.edu/?q=mfold

MutationTaster, http://mutationtaster.org/

NHLBI ESP Exome Variant Server, http://evs.gs.washington.edu/EVS/

OMIM, http://www.omim.org/

PolyPhen-2, http://genetics.bwh.harvard.edu/pph2/

SIFT, http://sift.jcvi.org/

## Supplementary material

Supplementary Material is available at *HMG* online.

Supplementary Data

Supplementary Data
