# Supplementary material for: Mutations and altered expression of SERPINF1 in patients with familial otosclerosis
Source: Hum Mol Genet. 2016 Apr 7;25(12):2393–403. doi: 10.1093/hmg/ddw106 (PMC5181625; doi:10.1093/hmg/ddw106)
Supplement: Supplementary Data [file supp_25_12_2393__index.html]

Mutations and altered expression of SERPINF1 in patients with familial otosclerosis — Mutations and altered expression of SERPINF1 in patients with familial otosclerosis — Supplementary Data 

# Mutations and altered expression of *SERPINF1* in patients with familial otosclerosis

## Supplementary Data

files

- Supplementary Data - pdf file
