# Supplementary material for: Mutations and altered expression of SERPINF1 in patients with familial otosclerosis
Source: Hum Mol Genet. 2016 Apr 7;25(12):2393–403. doi: 10.1093/hmg/ddw106 (PMC5181625; doi:10.1093/hmg/ddw106)
Supplement: Supplementary Data [file supp_ddw106_HMG-2016-D-00169_Ziff_Supplemental_Data.pdf]

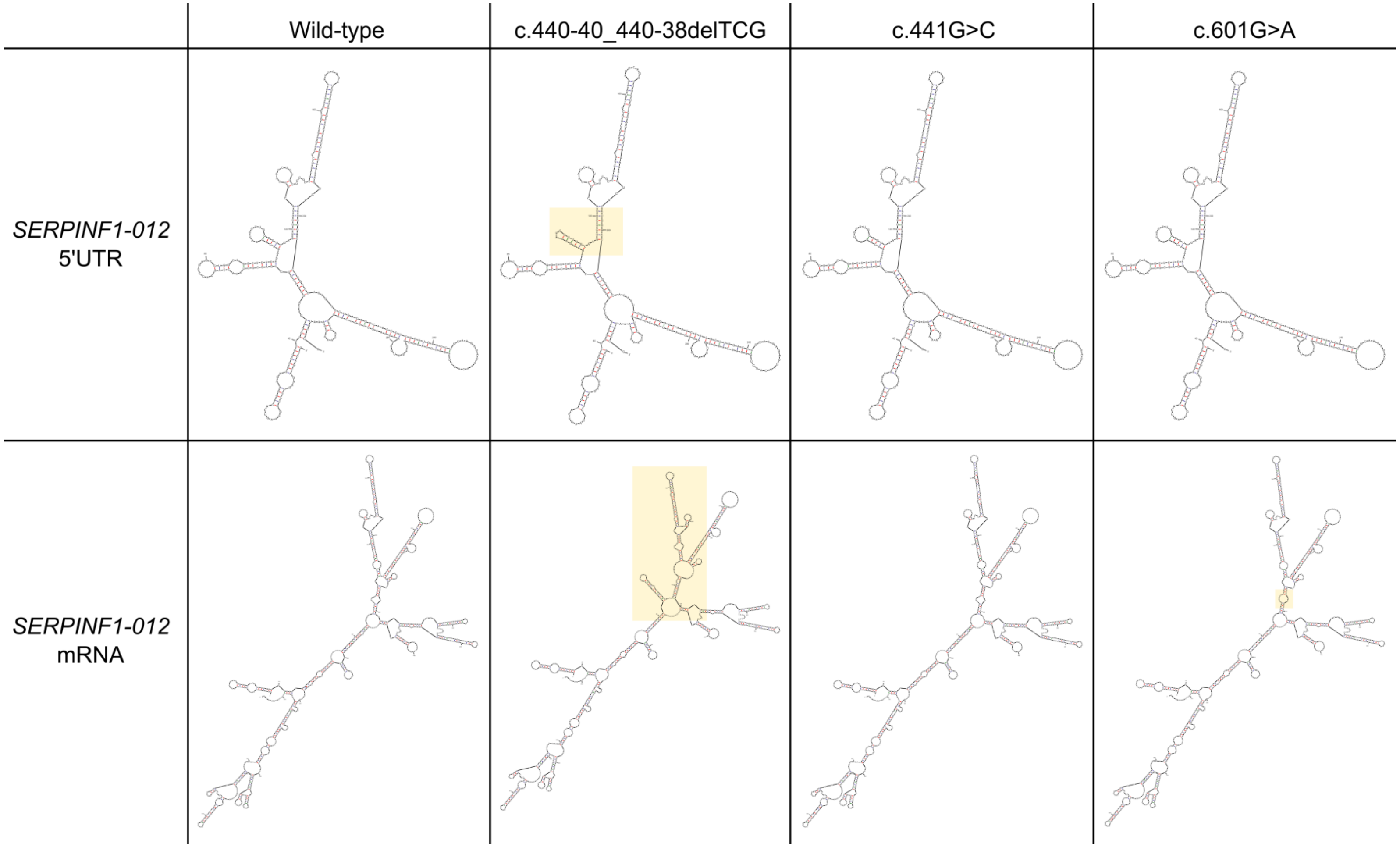

Figure S1. Predicted *SERPINF1-012* RNA foldings of 5'UTR variants. Putative RNA folding structures were predicted for the 5'UTR sequences only and the entire *SERPINF1-012* transcript (ENST00000573763). Comparisons were made between wild-type, c.440-40_440-38delTCG, c.441G>C and c.601G>A. Differences in mRNA structure compared to wild-type are highlighted (yellow box).

Table S1. PCR primers used to amplify *SERPINF1* exons for sequencing.

Primer	Direction	Gene	Sequence (5' to 3')	Amplicon
SERPINF1Exon2F	Forward	*SERPINF1*	AGTGACTAGCCCTGCCCAAC	309 bp
SERPINF1Exon2R	Reverse		AAGCCTGGCCTGGAACCT	
SERPINF1Exon3F	Forward	*SERPINF1*	CGTGAGGAGACAGTCCCTGT	345 bp
SERPINF1Exon3R	Reverse		TCAGCCACGTTTACGCAGAGG	
SERPINF1Exon4F	Forward	*SERPINF1*	GCCTACTTGGGCTCTCAGCAGA	311 bp
SERPINF1Exon4R	Reverse		ACATGCCTCAGGCAACTTGG	
SERPINF1Exon5F	Forward	*SERPINF1*	TGCTGAGCGCTAAACCAGAAC	323 bp
SERPINF1Exon5R	Reverse		AGAATTGGAGACGCGCTCACC	
SERPINF1Exon5bF	Forward	*SERPINF1*	AGATGCTGGCTGGGAAGTCAG	440 bp
SERPINF1Exon5bR	Reverse		CTGAGATCGCACCACTGCACTC	
SERPINF1Exon6F	Forward	*SERPINF1*	CTGACAGCTAAGCTCCCTTGA	369 bp
SERPINF1Exon6R	Reverse		TGTAGACAGCTGTCGGATCTCA	
SERPINF1Exon7F	Forward	*SERPINF1*	CTGGATGAAGGACGAGACCA	420 bp
SERPINF1Exon7R	Reverse		AGCCCTTGCGTTCTGCTTAGC	

Table S2. PCR primers used to amplify *SERPINF1* fragments and pGL4.10 construct for Gibson Assembly. Underlined sequences represent the overlapping region with the pGL4.10 vector nucleotides. Double underlined sequences represent the overlapping region with the *SERPINF1*-012 transcript nucleotides. Dotted underline indicates the c.601G>A variant in the additional primers.

Primer	Sequence (5' to 3')	Template	Amplicon
SERPINF1-012.FOR	GTACTGTTGGTAAAGCCACCGTCTCGCTCTGTTGCCCAG	*SERPINF1*-012 gDNA	811 bp
SERPINF1-012.REV	TTAATGTTTTTGGCATCTTCTGCTGATCTCATCGGGAATTTCCTTTGT		
pGL4-10.FOR	AATTCCCGATGAGATCAGCAGAAGATGCCAAAAACATTAAGAAGGGC	pGL4.10	4279 bp
pGL4-10.REV	CCTGGGCAACAGAGCGAGACGGTGGCTTTACCAACAGTACCG		
SERPINF1-012_601A.REV	TTAATGTTTTTGGCATCTTCTGCTGATCTCATTGGGAATTTCCTTTGT	*SERPINF1*-012 gDNA	811 bp
pGL4-10_601A.FOR	AATTCCCAATGAGATCAGCAGAAGATGCCAAAAACATTAAGAAGGGC	pGL4.10	4279 bp

Table S3. List of candidate genes and identified variants prioritised for followed-up in four families with otosclerosis.

Family	Gene	Variant	Segregated
A	*DNAH5*	c.7883T>G	N
	FRYL	c.227G>T	N
	PTK6	c.652A>T	N
	SMAP1	c.1224_1226dupCAT	N
B	*ACE*	c.3304C>A	N
	ANKS1A	c.1291C>A	Y
	CYP2D6	c.345delC	N
	HHIPL2	c.1300G>A	N
	LEPRE1	c.1626G>A	N
	MIA3	c.707G>A	N
	SERPINF1	c.601G>A	Y
	VPS53	c.107C>G	Y
C	*EGLN3*	c.496C>T	N
	ERCC6	c.2913G>C	N
	PDLIM5	c.842G>A	N
	TRIM17	c.915C>G	Y
D	*COL1A2*	c.808G>A	Y
	FOXK1	c.1384G>A	N
	FZD2	c.655C>T	Y
	GNGT1	c.82C>A	Y
	mir183	n.81G>T	Y
	SHANK2	c.2171C>T	N
	ZNF225	c.1698A>T	Y

Table S4. Predicted transcription factor binding sites in *SERPINF1-012* 5'UTR variants.

Nucleotide change	Transcription Factor	*SERPINF1*-012		Relevance
		Wild-type	Mutant	
c.440-40_440-38delTCG	PAX3	no binding	0.808	Regulates BMP-induced osteogenesis [36]
c.441G>C	HOXA9	0.855	0.855	
c.601G>A	NFKB1	0.933	0.933	
	DEAF1	0.907	no binding	
	POU3F2	no binding	0.866	
	IKZF1	0.936	0.993	
	RBPJ	no binding	0.951	Regulates osteoclastogenesis [37]
	HESX1	0.721	0.803	
	HMGA1	0.968	0.929	
	PRDM16	0.990	no binding	
	NFYA	no binding	0.936	Mediates regulation of bone sialoprotein [38]
	MAFK	0.853	0.853	
	CPHX	0.846	no binding	
